# Neuropsychological Sequelae and Neuroradiological Correlates of Arachnoid Cysts in Adults: A Systematic Review

**DOI:** 10.3390/brainsci16010103

**Published:** 2026-01-18

**Authors:** Odysseas Lorentzos, Panayiotis Patrikelis, Giuliana Lucci, Lambros Messinis, Stefanos Korfias

**Affiliations:** 1 Division Brain and Behavior, Laboratory of Neuropsychology and Behavioral Neuroscience, School of Psychology, Aristotle University of Thessaloniki, 54124 Thessaloniki, Greece; lorentzos@psy.auth.gr (O.L.); patrikelis@psy.auth.gr (P.P.);; 2 First Department of Neurosurgery, National & Kapodistrian University of Athens, 11527 Athens, Greece; 3 Independent Researcher, 00161 Rome, Italy

**Keywords:** arachnoid cysts, neuropsychological assessment, cognition, executive functions, functional neuroimaging

## Abstract

Background/Objectives: Intracranial arachnoid cysts (Acs) are congenital, usually benign lesions that are frequently regarded as clinically silent in adulthood. Nonetheless, growing evidence indicates that Acs may be associated with subtle but measurable cognitive dysfunction. This systematic review synthesizes neuropsychological and functional neuroimaging findings in adults with intracranial Acs, with a focus on cognitive profiles, functional interactions with the adjacent cortex, and postoperative reversibility. Methods: In accordance with PRISMA 2020 guidelines, MEDLINE/PubMed and Scopus were searched for English-language studies published up to 2023 that reported neuropsychological assessments and/or functional neuroimaging in adult patients with Acs, including single-case reports, case series, and group studies with pre- and post-operative data. Results: Sixty studies met the inclusion criteria. Across anatomical locations, Acs were most consistently associated with impairments in verbal and visual memory and learning, attention, and executive functions, as well as reduced processing or psychomotor speed, whereas language deficits were less consistently observed. Several studies reported postoperative improvement in one or more cognitive domains, suggesting partial reversibility in selected patients. Functional neuroimaging findings revealed altered cortical function in regions adjacent to the cyst, including reduced regional metabolism or cerebral blood flow and task-related activation changes, supporting a functional interaction between Acs and the neighboring cortex. Conclusions: Overall, adults with Acs may exhibit subtle cognitive alterations that vary according to cyst location and appear to be moderated by compensatory mechanisms. These findings underscore the clinical relevance of systematic neuropsychological evaluation and highlight the need for prospective, standardized studies integrating cognitive and neuroimaging outcomes.

## 1. Introduction

Intracranial arachnoid cysts (Acs) are congenital, benign malformations characterized by the accumulation of cerebrospinal fluid-like content within the arachnoid membrane [1]. They occur more frequently in males [2] and are most commonly located in the middle cranial fossa, with a predominance in the left hemisphere, while approximately two thirds develop supratentorially [1]. Although Acs may vary considerably in size and location, epidemiological studies estimate their prevalence in adults to range between 1.4% and 2.3% [3,4], with most cases identified incidentally through neuroimaging performed for unrelated reasons [5].

The pathogenesis of Acs is generally attributed to aberrant development of the subarachnoid space during embryogenesis [6], although alternative mechanisms, including abnormalities of the meninx primitive and early disturbances of brain development, have been proposed [6]. These hypotheses have been invoked to explain the frequent association of Acs with structural alterations such as temporal lobe hypoplasia or corpus callosum anomalies, particularly in intrahemispheric cysts [6]. Nevertheless, current evidence suggests that such associations are more likely coincidental than causally related [6]. Despite their often-considerable size and the marked displacement of adjacent cortical and subcortical structures, Acs are traditionally regarded as clinically silent throughout adult life [6,7]. When symptoms occur, they most commonly include headache, dizziness, seizures, or nonspecific neurological complaints [1,8], whereas cognitive symptoms are rarely reported as primary reasons for clinical referral. However, several studies have shown that subtle cognitive alterations may emerge during systematic neuropsychological evaluation, even in patients considered neurologically asymptomatic [9,10,11].

Over the past four decades, the literature addressing the neuropsychological consequences of Acs has progressively expanded. Owing to the relatively low prevalence of these lesions, most available evidence derives from single-case reports and small case series [12,13,14,15,16], although larger cohort studies—particularly those examining pre- and post-operative cognitive outcomes—have also been published [17,18,19,20,21]. Across studies, cognitive deficits have been described in multiple domains, including verbal and visual memory and learning, attention, executive functions, processing speed, and psychomotor performance, with considerable variability depending on cyst location and size [19,20].

The apparent discrepancy between extensive structural displacement and relatively preserved everyday functioning represents one of the most intriguing aspects of Acs. This observation has prompted questions regarding the extent to which cognitive dysfunction reflects direct effects of cortical compression, altered perfusion or metabolism of adjacent tissue, or long-term compensatory and adaptive mechanisms operating during neurodevelopment [22,23,24,25,26,27]. Functional neuroimaging studies have increasingly contributed to this debate by providing evidence of altered activation patterns, metabolic changes, and preserved or displaced functional organization in regions neighboring the cyst [24,25,26,27,28,29,30].

Against this background, the present systematic review aims to identify, synthesize, and critically evaluate neuropsychological and functional neuroimaging evidence in adults with intracranial arachnoid cysts. Emphasis is placed on cognitive profiles associated with different cyst locations, evidence of functional interaction between Acs and adjacent cortex, and the extent to which cognitive deficits may be reversible following surgical intervention.

## 2. Materials and Methods

### 2.1. Search Strategy

This systematic review was conducted in accordance with the PRISMA 2020 guidelines for reporting systematic reviews [29]. A comprehensive literature search was performed in the MEDLINE/PubMed(National Library of Medicine, Bethesda, MD, USA) and Scopus (Elsevier, Amsterdam, The Netherlands) databases to identify studies investigating neuropsychological functioning and functional neuroimaging findings in adults with intracranial arachnoid cysts (Acs).

The search strategy included articles published in English between January 1980 and December 2023. The following search terms were used in various combinations: “arachnoid cyst”, “cognition”, “cognitive”, “neuropsychological”, “memory”, “executive functions”, “attention”, “language”, “visuospatial”, “processing speed”, “functional neuroimaging”, and “reorganization”. Database-specific syntax was adapted as necessary. In addition, the reference lists of all included articles were manually screened to identify further relevant studies.

### 2.2. Eligibility Criteria

Studies were included if they met the following criteria:

(i) involved adult participants (≥18 years) diagnosed with intracranial arachnoid cysts; (ii) reported neuropsychological assessment, either as comprehensive testing or domain-specific evaluation, and/or functional neuroimaging data (e.g., fMRI, PET, and SPECT); (iii) were original peer-reviewed articles, including single-case reports, case series, and observational or interventional group studies; (iv) were published in English.

Studies focusing exclusively on pediatric populations were excluded, given the distinct neurodevelopmental implications in childhood ACs, except for mixed-age studies from which adult data could be clearly extracted. Articles addressing psychiatric manifestations without reporting at least minimal neuropsychological data were excluded. Studies in which ACs were considered secondary findings or consequences of other primary neurological disorders were also excluded.

### 2.3. Study Selection

**Figure 1:**
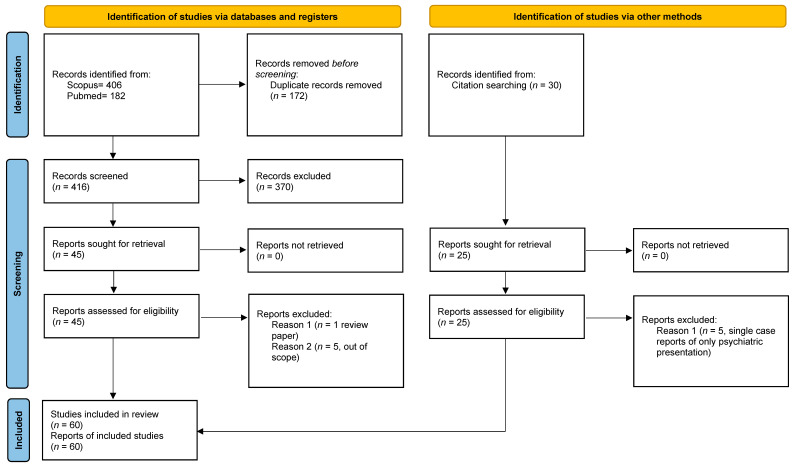
PRISMA 2020 flow diagram for the systematic review, which included searches of PubMed/Medline and Scopus electronic databases and citation searching.

Two reviewers (O.L. and P.P.) independently conducted the database search and screened titles and abstracts for eligibility. Duplicate records were removed using Mendeley Reference Manager. Full-text articles were subsequently assessed for inclusion based on the predefined criteria. Any discrepancies between reviewers were resolved through discussion and consensus. The study selection process is summarized in a PRISMA flow diagram (Figure 1).

### 2.4. Data Extraction and Synthesis

Data were extracted independently by the two reviewers using a standardized extraction form. Extracted variables included authorship, year of publication, study design, sample characteristics, cyst location and size (when reported), neuropsychological assessment tools, functional neuroimaging methods, type of intervention (if any), and cognitive or imaging outcomes. Given the substantial heterogeneity in study design, outcome measures, reporting standards, non-overlapping assessment tools and limited variance data, a qualitative narrative synthesis was adopted rather than a quantitative meta-analysis. To quantify recurrent patterns across cognitive domains, anatomical locations, and postoperative outcomes, descriptive quantitative summaries (frequency counts and proportions at the study level) were carried out in addition to narrative synthesis, without suggesting patient-level prevalence or causal inference.

### 2.5. Risk of Bias and Methodological Quality Assessment

The methodological quality and risk of bias of included studies were independently assessed by two reviewers (O.L. and P.P.). Case reports and case series were evaluated using the Joanna Briggs Institute (JBI) critical appraisal checklists [30,31], whereas observational group studies were assessed using the Newcastle–Ottawa Scale (NOS) [32]. Disagreements were resolved by consensus. Given the predominance of descriptive and exploratory study designs, risk of bias was considered primarily in the interpretation of findings rather than as a basis for study exclusion.

## 3. Results

### 3.1. Study Selection and Characteristics

The literature search identified a total of 59 studies meeting the inclusion criteria. Of these, 37 were single-case reports, 13 were case series or small cohort studies, and 10 were functional neuroimaging studies employing PET, SPECT, or fMRI methodologies (1 study was tabulated in both single-case reports and neuroimaging due to the nature of the study). The study selection process is illustrated in the PRISMA flow diagram (Figure 1).Risk of bias assessments for all included studies are provided in Supplementary Materials.

Across studies, substantial heterogeneity was observed with respect to sample size, cyst location, neuropsychological assessment protocols, and outcome reporting. The extracted study characteristics and key findings are summarized in Table 1, Table 2 and Table 3.

### 3.2. Single Case Reports

Thirty-seven single-case reports described neuropsychological findings in adults with intracranial arachnoid cysts [3,9,10,11,12,13,14,15,16,17,18,19,20,21,22,23,24,25,27,33,34,35,36,37,38,39,40,41,42,43,44,45,46,47,48,49,50,51,52,64,66]. Clinical presentation varied widely and included psychiatric symptoms (e.g., psychosis, mood disorders, and obsessive–compulsive symptoms), neurological complaints (e.g., seizures, headaches, and gait disturbance), cognitive complaints, or incidental findings identified through neuroimaging.

Neuropsychological assessment methods were highly variable. In several cases (24.3%), cognition was assessed only through brief screening instruments such as the Mini-Mental State Examination (MMSE) [12,35,38,45,49,66]. Other reports (29.7%) employed domain-specific neuropsychological tests or standardized batteries targeting memory, attention, executive functions, language, or visuospatial abilities [14,34,37,40,43,51,68]. A subset of studies (45.9%) reported comprehensive neuropsychological evaluations covering multiple cognitive domains [10,11,16,27,36,42,44,50,52].

**Figure 2:**
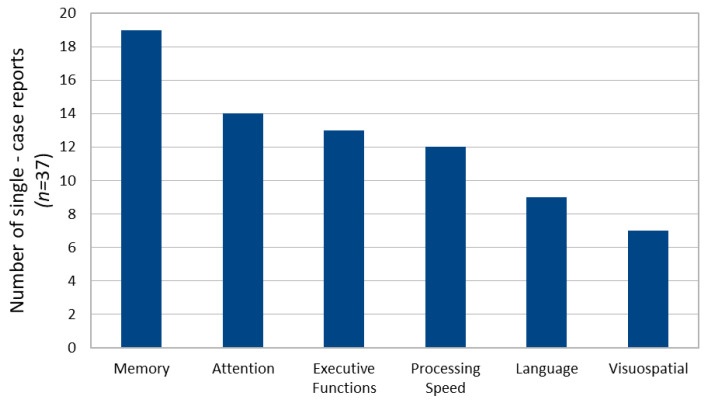
Frequency of cognitive deficits reported across neuropsychological domains in 37 single-case studies of adults with intracranial arachnoid cysts.

Across reports, cognitive deficits were most frequently documented in verbal, visual or episodic memory (51.4%), attention (37.8%), executive functioning (35.1%), processing speed and psychomotor performance (32.4%) see Figure 2. Language impairments were less consistently reported (24.3%) and were often subtle or task specific. In several cases (21.6%), cognitive dysfunction was observed in the absence of prominent neurological symptoms and was detected only through formal neuropsychological testing [16,27,48,52]. In single case reports descriptive cross tabulation suggested an anatomical pattern, where middle cranial fossa cysts were more often associated with memory and processing speed impairments and posterior fossa with executive and attention deficits. Frontal-only cases were few and showed heterogeneous profiles. Due to variation in reporting and assessments methods the findings are considered descriptive and hypothesis generating.

Post-intervention neuropsychological data were available in a subset of cases (32.4%), primarily following surgical decompression or shunting [9,15,19,21,36,39,41,46,50]. In these reports, partial or domain-specific cognitive improvement was frequently described (75.0%) and 25% remained unchained, although the extent and durability of improvement varied across cases.

Functional neuroimaging data reported in single-case studies included evidence of reduced regional cerebral metabolism, hypoperfusion, or electrophysiological slowing in cortical areas adjacent to the cyst [17,37,42,47,64]. These findings were observed both in surgically treated and conservatively managed cases. This synthesis consisted of heterogenous single-case studies. RoB was moderate to high due to descriptive designs, the lack of a control group and limited generalizability; however, the detailed presentation offered domain-specific insights.

### 3.3. Case Series and Group Studies

Thirteen studies employed case series or group-based designs to investigate neuropsychological functioning in adults with Acs [18,21,22,23,24,25,26,28,64,67,68,69,70]. Sample sizes ranged from small cohorts to larger observational studies, with most including pre- and post-operative assessments. Because cognitive outcomes were evaluated using a variety of instruments and domain definitions, cross-study aggregation of individual outcomes was methodologically incorrect, hence patient-level pooling across trials was not carried out.

**Figure 3:**
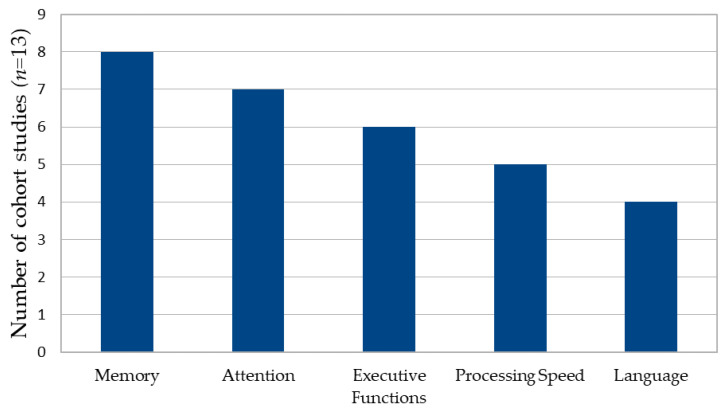
Frequency of cognitive deficits across domains in cohort studies of arachnoid cysts.

**Figure 4:**
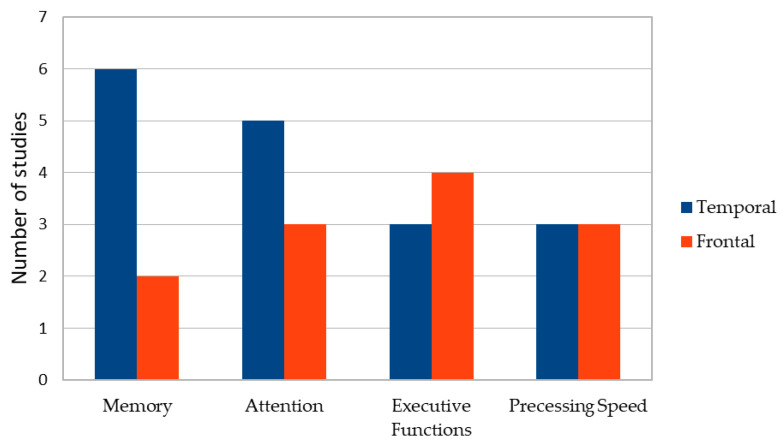
Cognitive deficit patterns by cyst localization.

Across studies, cognitive deficits were most consistently reported in memory (61.5%), attention (53.8%), executive functions (46.2%), processing speed (38.5%) and language (30.8%), with patterns varying according to cyst location and hemispheric dominance [22,23,24,25,26,64,70]. These patterns are illustrated in Figure 3. A descriptive contingency analysis at the study level demonstrated anatomical patterning of cognitive deficits (see Figure 4). Middle cranial fossa cysts (predominantly temporal) were frequently associated with memory (75%) and attentional impairments 62.5%, whereas frontal cysts were more commonly linked to deficits in executive functioning 80% and psychomotor speed 60%.

**Figure 5:**
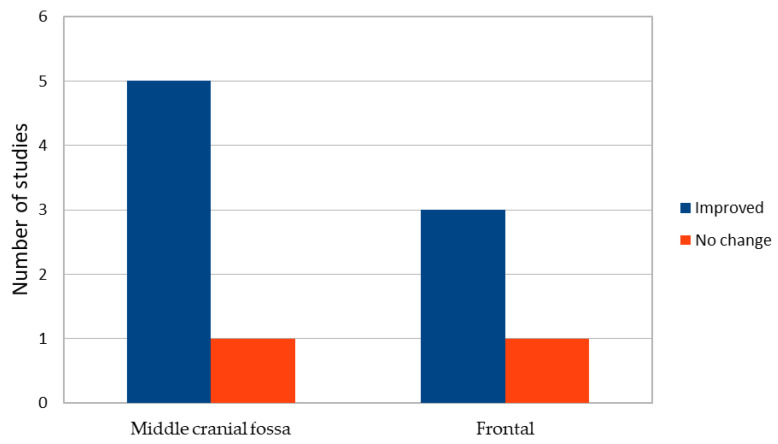
Postoperative cognitive outcomes by cyst localization.

Most studies (77.8%) documented postoperative cognitive improvement, particularly in memory, attention, executive functioning, and language-related tasks [18,22,25,26,28,64,67,69]. However, not all studies reported significant postoperative changes, and some (22.2%) failed to detect measurable cognitive differences between patients and healthy controls either before or after surgery [21,68] (see Figure 5).

Language-related outcomes were specifically investigated in dichotic listening and lateralization paradigms, with findings indicating altered auditory–verbal processing preoperatively and normalization following surgical intervention in some patients [24,67].

The case series and group study synthesis included small series and observational cohorts, mostly with pre- and post-operative neuropsychological data. The overall RoB was moderate, due to the sample size and non-randomization as well as heterogeneity in the outcome. Potential sources of heterogeneity among included studies were descriptively explored, by the grouping through cyst localization, cognitive domains, and surgical status, as presented above.

### 3.4. Functional Neuroimaging Studies

Ten studies employed functional neuroimaging techniques to investigate brain function in adults with intracranial Acs [22,23,24,25,26,27,28,68,69,70]. Imaging modalities included PET, SPECT, and fMRI, and study designs comprised both single cases and small case series.

Functional imaging findings most commonly demonstrated altered cortical activity, metabolism, or perfusion in regions adjacent to the cyst [22,23,24,25,26,70]. Reduced regional cerebral blood flow or glucose metabolism was frequently observed in frontal or temporal cortices neighboring the lesion, particularly in symptomatic patients [25,26,70].

Language and motor paradigms investigated through fMRI generally revealed preserved functional lateralization, despite marked displacement of cortical structures [23,24,68]. In some cases, evidence of bilateral activation or intrahemispheric reorganization was reported, particularly in motor and somatosensory networks [27,69].

Overall, functional neuroimaging studies indicated that Acs may be associated with localized functional alterations in adjacent cortex, while large-scale interhemispheric reorganization appeared to be uncommon.

The overall certainty in the presented evidence across domains was estimated as low to moderate. There was higher confidence for domains that were more consistently represented (e.g., memory, attention, executive functions). Conversely there was a lower degree of certainty for less consistently reported domains (language, processing speed). Functional neuroimaging data outcomes were moderate in certainty, since imaging findings were in accordance with neuropsychological evidence, despite the small sample size. RoB was moderate due to sample size and exploratory designs, alleviated, however, partly by the imaging outcome.

## 4. Discussion

The present systematic review synthesizes neuropsychological and functional neuroimaging evidence on adults with intracranial arachnoid cysts (Acs), challenging the long-standing view of these lesions as uniformly benign and clinically silent. Across a heterogeneous body of literature, the findings consistently indicate that Acs may be associated with subtle but measurable cognitive dysfunction, particularly when systematic neuropsychological assessment is employed.

### 4.1. Cognitive Profiles and Anatomical Correlates

Across study designs, cognitive impairments were most frequently reported in domains subserved by frontal and temporal networks, including verbal and visual memory, attention, executive functions, processing speed, and psychomotor performance. This pattern aligns with the predominant localization of Acs in the middle cranial fossa and frontal convexity, regions critically involved in higher-order cognitive processing [7,9,10]. Importantly, the observed deficits were often mild and domain-specific, rarely reaching a severity that would unequivocally compromise everyday functioning. This may partly explain why cognitive symptoms are infrequently reported as primary complaints and are instead uncovered during structured neuropsychological evaluation.

Language impairments were less consistently observed and, when present, tended to be task-dependent rather than reflecting overt aphasia. Findings from dichotic listening and functional imaging studies suggest that language lateralization is generally preserved despite substantial displacement of cortical tissue [22,23,67]. These observations argue against large-scale interhemispheric reorganization and instead support the notion of functional displacement or local adaptation within the affected hemisphere.

### 4.2. Evidence for Reversibility and Surgical Effects

A central question in the clinical management of Acs concerns the extent to which cognitive deficits are reversible. Several case reports and cohort studies documented postoperative cognitive improvement, particularly in memory, attention, executive functioning, and language-related tasks [10,11,18,19,20]. Such findings support a causal relationship between Acs and cognitive dysfunction, consistent with earlier proposals that symptom reversibility following surgical decompression provides indirect evidence of pathogenicity [7].

However, the literature is not uniform. Some studies failed to demonstrate significant cognitive change following surgery or reported comparable neuropsychological performance between patients and controls both pre- and post-operatively [3,21]. These discrepancies likely reflect differences in cyst characteristics, patient selection, baseline cognitive reserve, assessment sensitivity, and follow-up duration. Together, these findings suggest that reversibility is not universal but may depend on whether a critical threshold of functional compromise has been exceeded.

### 4.3. Functional Neuroimaging and Pathophysiological Mechanisms

Functional neuroimaging studies provide important insights into the mechanisms underlying cognitive alterations in Acs. PET and SPECT investigations frequently revealed reduced regional cerebral metabolism or perfusion in cortical areas adjacent to the cyst, particularly in frontal and temporal regions [25,26,70]. These findings support the hypothesis that cognitive deficits may arise from local hypoperfusion, metabolic disruption, or altered network efficiency, rather than from gross neuronal loss.

fMRI studies further demonstrated that, despite marked anatomical displacement, functional organization is often preserved, especially for language and motor systems [23,24,25]. In some cases, bilateral activation patterns or intrahemispheric reorganization were observed, particularly within motor and somatosensory networks [25,69]. Taken together, these data point to a complex interaction between structural deformation, vascular–metabolic factors, and neuroplastic adaptation.

### 4.4. Compensation, Cognitive Reserve, and Threshold Effects

One of the most salient themes emerging from this review is the apparent dissociation between structural abnormality and clinical expression. Large Acs may coexist with relatively preserved cognitive functioning, suggesting the operation of long-term compensatory mechanisms. This observation is consistent with a compensation–threshold model, whereby gradual developmental displacement allows adjacent networks to adapt, preserving function until compensatory capacity is saturated [71].

Once this threshold is exceeded—due to factors such as cyst growth, increased intracystic pressure, age-related vulnerability, or comorbid pathology—cognitive symptoms may emerge. This framework may also account for the variability in surgical outcomes, as decompression may restore function only when deficits are driven by reversible physiological mechanisms rather than by entrenched network reorganization.

### 4.5. Clinical and Research Implications

From a clinical perspective, the findings underscore the importance of systematic neuropsychological assessment in adults with Acs, even in the absence of overt neurological symptoms. Reliance on brief screening measures alone may underestimate subtle cognitive alterations that are nonetheless relevant for quality of life and functional outcomes. Additionally, the functional neuroimaging findings indicate that cyst size alone, does not reliably predict functional compromise or surgical necessity. The more subtle physiological mechanisms proposed may underlie both compromise and recovery of cognitive function and in conjunction with neuropsychological and clinical assessment inform surgical decision making.

From a research standpoint, the literature remains limited by methodological heterogeneity and a predominance of descriptive designs. Future studies would benefit from prospective, longitudinal approaches, standardized neuropsychological batteries, and multimodal neuroimaging protocols capable of capturing both functional and structural network changes over time. In particular, the role of white matter connectivity and large-scale network dynamics remains largely unexplored and represents a critical avenue for future investigation.

### 4.6. Limitations

Several limitations can be identified in the scope of the present review. Firstly, there was considerable variability in the methodologies of the included studies. The assessment protocol was not consistent among studies, with some being more thorough, while others were narrower in scope. Heterogeneity was also observed regarding sample sizes and study type, with many single-case reports or small cohorts limiting the overall generalization of the findings. The same holds for the type of intervention, if any, as well as the lack of consistent reporting of outcomes or follow-ups in some instances.

Additionally, the interpretation of improvement albeit comparable in terms of cognition in most small cohort studies was not always apparent between single-case studies, thus affecting comparability and practical significance of findings. The aforementioned factors constitute the reasons that a narrative review was adopted, since data heterogeneity could not allow for a quantitative comparison amongst most studies.

## 5. Conclusions

The majority of neuropsychological evidence on adults presenting with ACs points to aberrant cognitive functioning. The emerging pattern of cognitive impairment varies as a function of a cyst’s anatomical location. The reported deficits are varied, ranging from verbal and visual memory and learning, attention, executive functions, speed of information processing, expressive language, and psychomotor speed. Though traditionally viewed as silent, ACs and their presumed rapport with the adjacent cortex have been subject to careful neuropsychological scrutiny. Deficit reversibility following surgery further corroborates the above “interaction” hypothesis. A compensation–threshold mechanism is alluded to since cognitive changes are subtle and likely occur without being transferred to the other hemisphere. This review highlights the need for further research to understand the relationship between ACs and cognitive functions, considering the potential for brain adaptation and functional compensation over time. While ACs are often considered benign, their impact on cognitive functions warrants careful neuropsychological assessment.

## Figures and Tables

**Table 1:** Single case studies assessing cognition in Arachnoid Cysts.

Neuropsychological Outcome	Intervention	Neuropsychological Findings	Neuropsychological Tests	Cyst Location	Cyst Size	Clinical Presentation	Sex	Age	Study
Writing restored	Cyst excision	BDAE, word reading, token test, ROCFT, oral-written spelling, apraxia testing	Comprehensive neuropsychological assessment	Left Frontal Lobe	“Large”	APRAXIC AGRAPHIA	F	61	Hodges, 1991 [12]
Significant improvement was demonstrated in visual–perceptual abilities, constructional skill, verbal learning/memory, conceptual shifting, and psychomotor speed. Performance IQ	Fenestration of the cyst and cysto-peritoneal shunt	Expressive-language functions involving confrontation naming and verbal fluency, coordination speed, verbal learning and delayed, spontaneous recall of words, Facial recognition memory	Comprehensive neuropsychological assessment	Left temporal fossa, extending to the frontal and parietal region	15 × 3.5 × 2.5 cm	Incidental	M	20	Soukup et al., 1998 [13]
N/A	No	Visual information processing and memory dysfunction, word generation, inhibitory control, and motor function	Comprehensive neuropsychological assessment	Left Frontotemporoparietal	Galassi 3	Frontal headaches	M	33	Lebowitz et al., 2006 [14]
N/A	None	Processing speed, naming, executive attention	Comprehensive neuropsychological assessment	Left Anterior and Middle Cranial Fossa	16.4 × 7.7 cm	Complaints of memory impairment	M	65	Miskey & Gross, 2016 [15]
N/A	No	Processing speed Visuomotor processing speed, semantics and language, set shifting, abstract reasoning, problem solving, learning and memory for verbal information.	WASI, WTAR, TMT, SCWT, WCST, WMS-III, HVLT-R, BNT—Naming and Word Retrieval; COWAT Letter and Category Fluency Florida Token Test—Sentence Comprehension Benton Judgment of Line Orientation—Spatial Orientation Benton Facial Recognition Test—Meshulam’s Symbol Cancellation Test, Ideomotor and Ideational Praxis; Finger Tapping Test, Luria Motor Tests, BDI-II, GDS, GAS	Left Parietal, extending frontally occipitally, midline shift	Galassi 3	Symptoms and neuropsychological profile alluding to Corticobasal Degeneration	F	69	Dunn et al., 2012 [16]
Improvements in verbal initiation, articulation, word. Retrieval, and comprehension skills.	Outpatient course speech and communication therapy	Impaired word finding and impaired verbal fluency	Not fully specified	Left Dorsolateral Frontal	N/A	Speech changes	M	70	Bohnen et al., 2016 [27]
N/A	No	Autobiographical memory	WAIS, Corsi block, TMT A, WMS-R, German aphasia test	Left Temporal pole	Galassi 1	Diplopia, headache	M	45	Babinksy et al., 1994 [33]
N/A	Trifluoperazine	Visuo-motor perception and coordination, ability to appraise situations and comprehension of whole and part relationships, processing speed	MMPI, WAIS, SDMT	Left Frontotemporal	3.5 × 5 cm	Emaciation and dehydration, alexithymia and psychotic symptoms	M	40	Blackshaw & Bowen, 1987 [34]
MMSE (28/30)	Fenestration		MMSE (23/30), executive functions not specified	Left Frontotemporal	6.2 × 4.3 cm	Dizziness	F	81	Boomkens et al., 2010 [35]
3 years follow-up unchanged	No	Generalized cerebral dysfunction (verbal memory, visual attention, task-switching, and executive control, Reduced verbal fluency, Impairments in fine motor coordination, auditory discrimination and rhythm recognition)	MMSE (27/30), DRS, WAIS-R, Boston Naming Test, Verbal Fluency Tests—Category and Letter, CVLT, TMT A-B, Grooved Pegboard Test, Seashore Rhythm Test	Right Sylvian Fissure	N/A	Psychotic symptoms.	F	63	Cullum et al., 1994 [36]
N/A	No		MMSE (29/30)			Psychotic symptoms	M	21	Da Silva et al., 2007 [37]
N/A	No	No findings	MMSE	Right Middle Cranial Fossa	N/A	Alcohol dependence	M	26	Das et al., 2017 [38]
N/A	No	Cognitive inflexibility perseverations, processing speed, attention	SCWT, RAVLT, WCST, MMPI, Rorschach (mentioned, presumed comprehensive)	Left from the inferior to the vertex level.	5.5 × 10.5 × 12.5 cm	Headache, nervousness and attention problems.	F	44	Genis & Cosar, 2020 [39]
No reduction in psychiatric symptom severity	Cyst fenestration 4 years prior to assessment	No findings	WAIS-R subtests Similarities, Picture Completion and Block Design,	Posterior Fossa	Galassi 1–2	Pseudologia fantastica	F	52	Heidrich et al., 1996 [40]
Improvement in all WMS subscales	Cysto-peritoneal shunt	Verbal memory	WMS-R	Left Frontal Lobe	6.1 × 6.5 × 6.9 cm	Headaches, acute vertigo and memory disturbances	F	70	Kotil et al., 2007 [41]
Verbal fluency improved, MMSE	Cyst-peritoneal shunt	Dysexecutive syndrome, selective Attention and deficit of free recall in episodic memory,	Comprehensive neuropsychological assessment	Right frontal	9.5 × 6.5 × 5.8 cm	Gait and cognitive slowing	F	75	Mormont et al., 2016 [42]
N/A	No	No findings	Clock Drawing Test, MMSE, Three Objects Three Places Test, emotion recognition, face discrimination, famous faces identification,	Right-anterior Temporal	Galassi 2	Capgras delusion	M	87	Nuara et al., 2020 [43]
N/A	No	Fine motor speed	Comprehensive neuropsychological assessment	Left-sided arachnoid cyst located in the middle cranial fossa	“Large”	Violent Behavior	M	65	Paradis et al., 1994 [44]
N/A	No	Auditory-verbal memory and learning (RALVT, BSRT), selective attention, divided attention	Comprehensive neuropsychological assessment	Left Frontotemporal	11 × 4 cm	Trigeminal neuralgia, subdural/epidural hematoma superimposed to the cyst, subjective memory complaints	M	50	Patrikelis et al., 2022 [45]
Resolution of expressive aphasia and mild improvement in cognitive function (measurement not specified)	Microsurgical excision		MMSE (20/30)	Left Parietal Lobe	6.7 × 5.3 cm	Difficulty with speaking for 1 month	F	56	Raj et al., 2018 [46]
N/A	No	None	“Formal and extensive”	Middle cranial fossa	Large	Transient global amnesia	M	60	Stracciari et al., 1987 [47]
N/A	Sertraline, haloperidol	None	MMSE (29/30)	Left posterior fossa	2.6 × 1.5 cm	Obsessive–compulsive disorder	F	62	Tonna et al., 2014 [48]
Improvement in all WMS-R subscales	Cyst excision	Verbal memory	WMS-R	Left frontal convexity	N/A	Memory disturbance	F	48	Tsurushima et al., 2000 [49]
N/A	No	Attention, information processing, verbal and visuospatial attention, working memory, planning, problem solving, encoding of information across all memory tasks, recognition and inhibitory control.	NIMHANS Neuropsychology Battery	Retro-cerebellar	4.2 × 3.2 cm	Schizophrenia, later OCD	M	45	Varshney et al., 2020 [50]
N/A	No	Verbal memory	Comprehensive neuropsychological assessment	Left frontotemporal	Galassi 3	Alzheimer’s disease	M	66	Wahl et al., 2019 [51]
Expressive speech, verbal fluency, psychomotor speed, bilateral fine motor speed, and mood. Memory verbal fluency unimproved.	Cyst fenestration	Expressive language, cognitive flexibility, bilateral manual dexterity, psychomotor slowing, attention	Not specified presumed comprehensive	Left orbital apex	Galassi 1	Left-sided headache, double vision, and left-sided facial numbness, aphasia	F	49	Zwagerman et al., 2016 [52]
Improved RTs in vigilance	Fenestration (frontal only)	Vigilance, reaction time	Wiener Test System	Frontal and Temporopolar	1.5 × 2.5 × 5 cm (frontal) and 3 × 1.5 × 2 cm (temporopolar)	Emotionally unstable personality disorder	M	20	Bechter et al., 2010 [53]
N/A	No	Phonemic and category switching fluency, digit span forward and backwards, cube draw and copy, and affect, with preservation of verbal learning and recall	Cerebellar Cognitive Affective/Schmahmann Syndrome Scale	Retro-cerebellar		Neurodevelopmental and psychiatric symptoms	M	32	Guell et al., 2020 [54]
Improvement in mental control and concrete and abstract thinking;	No (clozapine 125 mg/day, lamotrigine 100 mg/day and diazepam)	Logical thinking, learning ability and mental control, as well as impairment in executive functions, His intellectual efficacy, psychomotor speed and memorizing ability were decreased to the level of mild intellectual disability, and his concrete and abstract thinking to the level of moderate intellectual disability.	BETA-II, WMS, Cornell index, WDCT, Mosaic Test, WCST	Posterior cranial fossa.	6.3 × 5.9 × 4.4 cm	Imperative auditory hallucinations	M	22	Škarić et al., 2021 [55]
N/A	No	Deficits on measures of language, visuoperception, memory, and abstract reasoning in the context of relatively intact auditory attention and working memory	WAIS, RBANS	Left cerebral convexity	“Large”	Overdose admission	F	68	Yunes & Posada, 2021 [56]
Psychotic symptoms resolved	Risperidone	None reported	MMSE (27/30)	Anteromedial aspect of middle cranial fossa	3 × 2.5 × 2 cm	Psychosis	M	57	Bahk et al., 2002 [57]
Improvement, outpatient treatment	No (valproic acid and atypical neuroleptic medication)	Attention deficits, including short-term and working memory. Visual perception/visual construction	Not specified	Left temporal pole, with extension to the insula cisterna	N/A	Bipolar disorder	F	51	Claussen et al., 2013 [58]
Not reported	Not reported	No findings	Not specified	Right Anterior Temporal Lobe (Uncus)	1 cm	Depression With psychotic features.	Female	58	Cohen, 1989 [59]
N/A	N/A	None	MMSE (30/30)	Right Anterior Temporal and Right Lateral Prefrontal	7.6 × 4 × 8.1 cm	Major depression	M	58	Deseilles et al., 2009 [60]
N/A	No	Stroop test	Presumed comprehensive	Left Temporal Fossa	N/A presumed large	Psychosis	M	32	Lanczik et al., 1989 [61]
N/A	No, amitriptyline	Sustained attention and working memory, and on learning, sequencing and switching, mild perseveration and “disinhibition”, visuospatial reconstruction	Comprehensive neuropsychological assessment	Midline Cerebellum	N/A	Pathological crying, ataxia	M	70	Parvizi & Schiffer, 2007 [62]
N/A	Microsurgical cystostomy	No findings	MMSE, FAB, MOCA, TMT A-B	Right temporal region	7 × 8 × 7 cm	Everyday intensive headaches in the right temporal region	M	N/A	Stanishevskiy et al., 2021 [63]

Note: M, Male; F, Female; N/A: data not available, BDI-II, Beck Depression Inventory-II; BNT, Boston Naming Test; COWAT, Controlled Oral Word Association Test; CVLT, California Verbal Learning Test; DRS, Dementia Rating Scale; FAB, Frontal Assessment Battery; GAS, Geriatric Apathy Scale; GDS, Geriatric Depression Scale; HVLT-R, Hopkins Verbal Learning Test Revised; MMPI, Multiphasic Minnesota Personality Inventory; MMSE, Mini-Mental State Examination; MOCA, Montreal Cognitive Assessment; RBANS, Repeatable Battery for the Assessment of Neuropsychological Status; RAVLT, Rey Auditory Verbal Learning Test; ROCFT, Rey–Osterrieth Complex Figure Test; SCWT, Stroop Color-Word Test; SDMT, Symbol Digit Modalities Test; TMT A-B, Trail Making Test A-B; WAIS, Wechsler Adult Intelligence Scale; WASI, Wechsler Abbreviated Scale of Intelligence; WMS-R/III, Wechsler Memory Scale Revised/III; WCST, Wisconsin Card Sorting Test; WDCT, Wartegg Drawing Completion Test; WTAR, Wechsler Test of Adult Reading.

**Table 2:** Case study series assessing cognition in Arachnoid Cysts.

Neuropsychological Outcome in Operated Group	Operated	Neuropsychological Findings	Neuropsychological Tests	Cyst Location	Cyst Size	Clinical Presentation	Sex	Age	N	Study
Improvement, ROCFT, MMSE, SRB, COWAT	Y	Confrontational naming, Figure copy	BNT, ROCFT, DSTB, MMSE, COWAT	17 left temporal, 2 right temporal, 1 parietal left	4 × 2 × 2 to 8 × 6 × 6 cm	Headache 12, Dizziness 1	10 F, 11 M	40.1	21	Agopian-Dahlenmark et al., 2020 [20]
Color-Word Interference inhibition/switching, color naming, Verbal Fluency test, letter fluency; condition 2, category fluency; condition 3, category switching; condition 4, total switching accuracy Tower test	Y	SCWT, Verbal Fluency, Tower test (compared to normal controls)	D-KEFS (Color-Word Interference test, Verbal Fluency test, and Tower test)	14 left, 8 right, 19 temporal fossa, 3 frontal	Galassi type I; *n* = 9, Galassi type II to III; *n* = 10	Headache and Dyscognition	10 F, 12 M	43	22	Gjerde et al., 2013 [11]
Improvements in visual attention: reduced RTs and subjective reports; no change in response accuracy	Y	Prolonged RTs pre-surgery, right hemisphere cysts impaired attention shift, left hemisphere cysts impaired visual search.	Posner cue-target paradigm and visual search paradigm	21 temporal 6 frontal	Gallasi I–III	N/A	10 F, 17 M	41	27	Gundersen et al., 2006 [18]
Improvement in time navigating through the labyrinth and in errors	Y	Same number of errors, longer time navigating	labyrinth test	temporal fossa: 31 left 14 right	17 cysts type Galassi I, 22 Galassi type II and 6 as larger than type Galassi II (type III or II–III)	Dyscognition, Epilepsy, Headache, Vertigo	26 M, 19 F	41.3	45	Isaksen et al., 2013 [64]
Psychiatric symptom resolution post-surgery in operated cases; pharmacological treatment for others.	2 patients	Three cases (n. 4, 5, 8) showed evidence of “frontal lobe” deficits in executive and self-regulatory behaviors. One patient (Case 3) had an I.Q. in the intellectually disabled range, and one (Case 8) was in the borderline range.	Pfeiffer Mental Status Exam, Wechsler Memory Scale, Benton Word fluency, WCST, Boston Naming Test, Hooper Visual Organization Test, Greek Cross and Drawings, WAIS	right parietal-occipital, right temporal, right frontal, right basal ganglia, left thalamus, left temporal, quadrigeminal plate		Psychiatric symptoms: delusions, hallucinations, psychosis, Tourette’s, conversion disorder	6 F, 2 M		8	Kohn et al., 1989 [65]
Only some improved	14 patients	Most patients performed within normal ranges; memory disturbances and initiative deficits in seizure cases; no direct correlation between cyst side and cognitive deficits; some patients had mild language or motor delays in childhood.	WAIS, ROCFT, BVRT		18 left-sided, 10 right-sided	Seizures (13 cases), Headache, Hemiparesis, Dyslexia, Delayed speech development, Stuttering.	6 F, 22 M	5 to 59 (M = 28)	28	Kunz et al., 1988 [66]
N/A	N/A	psychomotor reduction, reduced capacity for information processing and a difference between fluid and premorbid intelligence, memory	vocabulary-test, information processing. LGT-3 (Learning and Memory Test), verbal and non-verbal. Attention capacity and psychomotility were measured (Aufmerksamkeits-Belastungstest, d2,; Wiener Determinations-Test), MMPI	-	9 cysts in medial cranial fossa (3 extending to anterior cranial fossa), 1 cyst in paramedian and basal left anterior cranial fossa	Headache, Temporal Lobe Epilepsy Abnormalities in behavior	10 M	21–26	10	Lang et al., 1985 [9]
No significant post-operative differences	Y	None	MMSE, Bingley visual memory, Identical Forms, RAVLT, ROCF, Swedish Stroop, Grooved Pegboard, Target Reaction Time.	16 temporal 9 frontal 3 parietal 5 occipital 13 posterior fossa 1 suprasellar 1 intraventricular 1mesencephalic 1ambient cistern	49.5 (52.4) mL	Headache, Dizziness or Imbalance, Trauma, Visual disturbance, Seizures, Cognitive impairment, Focal neurological signs	57 F, 68 M	43	125	Rabiei et al., 2018 [21]
N/A	N	No differences in MMSE or MADRS scores between subjects with cysts and controls; cysts were incidental findings with no cognitive or psychological impact.	MMSE		Galassi type I	No significant difference in headache, dizziness, cognitive impairment, depression, dementia, epilepsy, or previous head trauma between cases and controls.	24 F, 5 M	76.7	29	Rabiei et al., 2016 [3]
All tests	Y	All tests lower relative to control group	BVRT, Street Gestalt Test, SCWT, TMT A-B	temporal (43 patients; 28 left, 15 right), frontal (11 patients; 10 right, 1 left), and right parieto-occipital (1 patient; right)		Headache (48 patients), Dizziness/Nausea (9 patients), Epilepsy (8 patients)	17 F, 38 M	Median 36/37	55	Raeder et al., 2005 [10]
Inhibition, switching	Y		CANTAB: PAL and DMS assessed temporal lobe functions, while SOC and IED	Middle fossa, unilateral (left: 14, right: 8),	Galassi types I–III	Headache (77.9%), dizziness (24.8%), Dyscognition, Seizures.	9 F, 13 M	42.9	22	Torgersen et al., 2010 [19]
dichotic perception and memory	Y	Better LE recall than RE recall, forced attention	DMT, DLT	Left Temporal fossa	Most Galassi II to III, 2 Galassi I	Headache, Epilepsy, Hemiparesis	2 F, 11 M		13	Wester & Hugdahl, 1995 [17]
postoperative improvement in verbal laterality and cognitive function; normalization of REA in patients with left temporal and frontal cysts.	Y	Preoperative DL test: 51% REA, 39% LEA, 10% NEA; Postoperative DLT: 73% REA, significant cognitive normalization after surgery	DLT with consonant-vowel syllables, Raczkowski Handedness questionnaire.	34 left (6 frontal) 12 right (3 frontal)	Temporal cysts: 11 Type I, 15 Type II, 5 Type III (left); 5 Type I, 4 Type II, 2 Type III (right);	Headache, Cognitive suppression, Language and Memory impairment (preoperatively) No pathological left-handedness observed.	24 F, 27 M		51	Wester & Hugdahl, 2003 [67]

Note: M, Male; F, Female; N/A: data not available; Y: Yes; N: No BVRT, Benton Visual Retention Test; CANTAB, Cambridge Neuropsychological Test Automated Battery; COWAT, Controlled Oral Word Association Test; DLT, Dichotic Listening Test; DMS, Delayed Matching to Sample; DMT, Dichotic memory task; DSTB, Dureman Salde Test Battery; IED, Intra-Extra Dimensional; LEA, Left Ear Advantage; LE, Left Ear; MADRS, Montgomery–Åsberg Depression Rating Scale; MMSE, Mini-Mental State Examination; NEA, No Ear Advantage; PAL, Paired Associate Learning; REA, Right Ear Advantage; RE, Right Ear; ROCFT, Rey–Osterrieth Complex Figure Test; SOC, Stockings of Cambridge; SCWT, Stroop Color-Word Test; SRB, Selective Reminding Test; TMT A-B, Trail Making Test A-B.

**Table 3:** Neuroimaging findings in single case studies or case study series in subjects with Arachnoid Cysts.

Observed Deficits	Neuropsychological Tests Used	Key Findings	Clinical Presentation	Cyst Location	Age	Sample Size (N)	Study
No pre-surgery deficits; post-surgery showed cortical reorganization	fMRI and motor functional tests	Reorganized motor control pathways	Asymptomatic but cortical displacement	Left Frontal	36	1	Alkhadi et al., 2003 [25]
Apraxia and aphasia prior to treatment	Verbal and motor neuropsychological tests	Focal glucose hypometabolism near cyst	Motor speech apraxia and aphasia	Dorsolateral Left Frontal	70	1	Bohnen et al., 2016 [27]
Improved post-surgical cognitive deficits	fMRI and cognitive tests post-surgery	Improvement post-surgery with fMRI changes	Headache, vertigo, and limb weakness	Right Sylvian Fissure	45	1	Caruso & Colonese, 2006 [68]
ACC hyperactivation during panic stimuli	fMRI	ACC activation on fMRI during panic stimuli	Panic disorder with agoraphobia	Right Temporal	33	1	de Melo Neto et al., 2009 [69]
None observed	None	Normal glucose metabolism in adjacent cortex	Asymptomatic	Right Frontal	51	1	Hubele et al., 2013 [70]
No observed language lateralization deficits	Language dominance tested via fMRI	Language lateralization unaffected by cysts	Language dominance maintained despite displacement	Left Temporal Fossa	19–51	5	Hund–Georgiadis et al., 2002 [23]
Adult patients showed cognitive normalization post-surgery	SPECT with rCBF for adult subset	Significant rCBF improvement post-treatment; cognitive functions normalized in adults	Headache, cognitive decline	Sylvian Fissure	18–42	6	Martínez-Lage et al., 2006 [26]
Impaired rCBF prior to treatment; normalized post-surgery	SPECT with rCBF assessment	Impaired rCBF normalized post-treatment	Headache, developmental delay, seizures	Sylvian Fissure	2–42	11	Martínez-Lage et al., 2006 [26]
None observed	None	Functional motor cortex displacement with normal function	Motor cortex displacement without symptoms	Right Hemispheric	66	1	Nickel et al., 2007 [24]
No observed deficits in language processing	fMRI for language localization	Language localized without interhemispheric reorganization	No significant language reorganization	Left Temporal	-	4	Stowe et al., 2000 [22]
Mild cognitive decline (specifics not reported)	Neuropsychological tests (unspecified)	Cortical plasticity preserved despite large cyst	Headache and mild cognitive decline	Frontal Convexity	38	1	Taskapilioglu et al., 2017 [28]

Note: ACC, Anterior Cingulate Cortex; fMRI, functional Magnetic Resonance Image; rCBF, regional Cerebral Blood Flow; SPECT, Single Photon Emission Computed Tomography.

## Data Availability

No new data were created or analyzed in this study.

## Supplementary Materials

The following supporting information can be downloaded at: https://www.mdpi.com/article/10.3390/brainsci16010103/s1.
