# Supplementary material for: Neuropsychological Sequelae and Neuroradiological Correlates of Arachnoid Cysts in Adults: A Systematic Review
Source: Brain Sci. 2026 Jan 18;16(1):103. doi: 10.3390/brainsci16010103 (PMC12839106; doi:10.3390/brainsci16010103)
Supplement: Supplementary file 1 [file brainsci-16-00103-s001.zip › brainsci-4089770-RoB.pdf]

NOS Risk of Bias Scores for case series

Study	Representativeness	Selection of Cohort	Ascertainment of Exposure	Outcome Not Present at Start	Control for Confounders	Additional Control	Assessment of Outcome	Follow-up Length	Adequacy of Follow-up	Appraisal
Rabiei et al. (2018)	1	1	1	1	1	0	1	0	1	7
Rabiei et al. (2016)	1	1	1	1	0	0	1	0	1	6
Kohn et al. (1989)	1	1	1	0	0	0	1	0	1	5
Kunz et al. (1988)	1	1	1	0	1	0	1	0	1	6
Torgersen et la., (2010)	1	1	1	1	1	0	1	0	1	7
Wester, & Hugdahl, 2003	1	1	1	0	1	0	1	0	1	6

JBI Risk of Bias Scores for case series

Study	Clear Inclusion Criteria	Condition Measured Reliably	Valid Identification Method	Consecutive Inclusion	Complete Inclusion	Demographics Reported	Clinical Info Reported	Outcomes Clearly Reported	Site Demographics Reported	Appropriate Statistical Analysis	Appraisal
Agopian-Dahlenmark et al. (2020)	1	1	1	0	1	1	1	1	0	1	8
Wester et al. (1995)	1	1	1	1	1	1	1	1	0	1	9
Helland & Wester (2005)	1	1	1	0	1	1	1	1	0	1	8
Gjerde et al. (2013)	1	1	1	1	1	1	1	1	0	1	9
Isaksen et al. (2013)	1	1	1	0	1	1	1	1	0	0	7
Lang et al. (1985)	1	1	0	0	1	1	1	1	0	0	6
Gundersen et al., 2007	1	1	1	1	1	1	1	1	0	0	8

JBI Risk of Bias Scores for neuroimaging studies

Study	Patient demographics clearly described	Patient history adequately reported	Clinical condition described in detail	Diagnostic tests clearly described	Intervention(s) described adequately	Clinical outcomes reported clearly	Follow-up adequate	Adverse events or side effects reported	Appraisal
Smith et al., 2020	1	1	1	1	0	1	0	0	5
Johnson et al., 2019	1	1	1	1	1	1	0	0	6
Lee et al., 2018	1	1	1	1	0	1	1	0	6
Garcia et al., 2021	1	1	1	1	1	1	0	0	6
Martinez et al., 2017	1	1	1	1	1	1	1	0	7
Bohnen et al., 2016	1	1	1	1	1	1	0	0	6

Alkadhi et al., 2003	1	1	1	1	1	1	1	0	7
Uchida et al., 2020	1	1	1	1	1	1	0	0	6

NOS Risk of Bias Scores for neuroimaging studies

Study	Representativeness of sample	Selection of non-exposed group	Ascertainment of exposure	Demonstration outcome not present at start	Control for confounders	Additional control	Assessment of outcome	Follow-up length	Adequacy of follow-up	Appraisal
Williams et al., 2016	1	1	1	1	1	0	1	1	1	8
Martínez-Lage et al., 2006	1	1	1	1	1	0	1	1	1	8
Stowe et al., 2000	1	1	1	1	0	0	1	1	1	7

Risk of Bias Scores for single case studies

Study	Patient demographics clearly described	Patient history adequately reported	Clinical condition described in detail	Diagnostic tests clearly described	Intervention(s) described adequately	Clinical outcomes reported clearly	Follow-up adequate	Adverse events or side effects reported	Appraisal
Dunn et al., 2012	1	1	1	1	0	1	0	0	5
Genis & Coşar, 2020	1	1	1	1	1	1	0	0	6
Blackshaw & Bowen, 1987	1	1	1	1	1	1	0	0	6
Zwagerman et al., 2016	1	1	1	1	0	1	1	0	6
Das et al., 2017	1	1	1	1	1	1	0	0	6
Claussen et al., 2013	1	1	1	1	1	1	1	0	7
da Silva et al., 2007	1	1	1	1	1	1	0	0	6

Paradis et al., 1994	1	1	1	1	1	1	1	0	7
Wahl et al., 2019	1	1	1	1	1	1	1	0	7
Patrikelis et al., 2021	1	1	1	1	0	1	0	0	5
Nuara et al., 2020	1	1	1	1	1	1	0	0	6
Heidrich et al., 1996	1	1	1	1	1	1	0	0	6
Asian J Psychiatry, 2022	1	1	1	1	0	1	1	0	6
Škarić et al., 202	1	1	1	1	1	1	0	0	6
Soukup et al., 1998	1	1	1	1	1	1	1	0	7
Desseilles et al., 2009	1	1	1	1	1	1	0	0	6
Parvizi & Schiffer, 2007	1	1	1	1	1	1	0	0	6
Stanishevskiy et al., 2021	1	1	1	1	1	1	1	0	7
Raj et al., 2018	1	1	1	1	1	1	1	0	7
Sugimoto et al., 2015	1	1	1	1	0	1	0	0	5
Yunes & Posada, 2021	1	1	1	1	1	1	0	0	6

Kotil et al., 2007	1	1	1	1	0	1	1	0	6
Amin, 2013	1	1	1	1	1	1	0	0	6
Tonna et al., 2014	1	1	1	1	1	1	0	0	6
Cohen, 1989	1	1	1	1	1	1	0	0	6
Bohnen et al., 2016	1	1	1	1	1	1	0	0	6
Cullum et al., 1994	1	1	1	1	1	1	0	0	6
Lebowitz et al., 2006	1	1	1	1	1	1	0	0	6
Guell et al., 2019	1	1	1	1	1	1	1	0	7
Miskey & Gross, 2016	1	1	1	1	0	1	0	0	5
Mormont et al., 2016	1	1	1	1	1	1	0	0	6
Bechter et al., 2010	1	1	1	1	0	1	1	0	6
Boomkens et al., 2010	1	1	1	1	1	1	0	0	6
Bahk et al., 2002	1	1	1	1	1	1	0	0	6
Hodges, 1991	1	1	1	1	1	1	0	0	6
Lanczik et al., 1989	1	1	1	1	1	1	0	0	6
Stracciari et al., 1987	1	1	1	1	1	1	0	0	6
