# Supplementary material for: Neuropsychological Sequelae and Neuroradiological Correlates of Arachnoid Cysts in Adults: A Systematic Review
Source: Brain Sci. 2026 Jan 18;16(1):103. doi: 10.3390/brainsci16010103 (PMC12839106; doi:10.3390/brainsci16010103)
Supplement: Supplementary file 1 [file brainsci-16-00103-s001.zip › brainsci-4089770-Search Terms.pdf]

Search Terms Used

Scopus

(TITLE-ABS-KEY("arachnoid cyst" OR "arachnoid cysts" OR "intracranial arachnoid cyst"))

AND

(TITLE-ABS-KEY("cognition" OR cognit* OR "mental processes" OR "executive function" OR "memory" OR "language" OR "visual perception" OR "visuospatial" OR "attention" OR "neuropsychology" OR neuropsychol OR “functional neuroimaging”))

Pubmed

(("arachnoid cyst" OR "arachnoid cysts" OR "intracranial arachnoid cyst" OR "intracranial arachnoid cysts")

AND

("cognition" OR cognit* OR "mental processes" OR "executive function" OR "memory" OR "language" OR "visual perception" OR "visuospatial" OR "attention" OR "neuropsychology" OR neuropsychol OR "functional neuroimaging" OR "reorganization"))
